# Feasibility of continuous exhaled breath analysis in intubated and mechanically ventilated critically ill patients

**DOI:** 10.1186/cc9563

**Published:** 2011-03-11

**Authors:** LD Bos, PJ Sterk, MJ Schultz

**Affiliations:** 1 Academic Medical Center, Amsterdam, the Netherlands

## Introduction

Pulmonary elimination of volatile molecules (so-called volatile organic compounds (VOCs)) is altered in a variety of pulmonary and nonpulmonary diseases [1]. Because breath of intubated and mechanically ventilated critically ill patients is continuously available, detection of changes in VOC patterns could be used to monitor these patients. We hypothesized that an electronic nose (eNose) provides a reliable and continuous read-out of changes in patterns of exhaled VOCs (so-called breathprints).

## Methods

An observational pilot study of six intubated and mechanically ventilated critically ill patients. Breathprints were collected by means of an eNose every 10 seconds for ± 7 hours. The patient sample size is too small for statistical analysis between patients, but varying fluctuations could be analysed within each patient.

## Results

**Figure 1:**
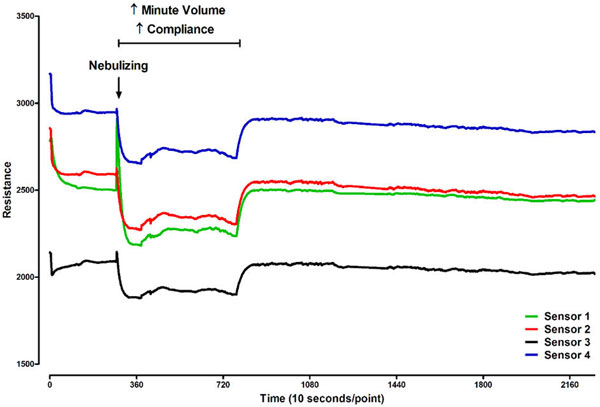
**Changes in resistance of sensors during 7 hours of mechanical ventilation**.

Breathprints fluctuated considerably over time (SEM 1.18). However, typical changes could be detected: for example, salbutamol inhalation, decreased static compliance and increased minute volumes all caused a rapid change in the breathprints (illustrated in Figure 1).

## Conclusions

Continuous monitoring of exhaled breath using an eNose is feasible in intubated and mechanically ventilated patients. Our data suggest that changes of breathprints within patients could be used to assess the clinical course of the patients.
